# Miscellaneous

**Published:** 1897-12

**Authors:** 


### MISCELLANEOUS.

### HYDROZONE FOR DISORDERS OF THE GENITO-URINARY TRACT.

Dr. John Aulde, of Philadelphia (*Medical Times and Register* of Philadelphia, Pa., December 5, 1896), states that about eight years ago he was forcibly impressed with the value of peroxide of hydrogen in a protracted case of gonorrhea. The disease had persisted for three months despite the treatment of several attendants, there being a constant discharge, and in addition there was an orchitis present, the left testicle being about as large as a baseball. Treatment consisted of the local use of injections of equal parts of peroxide of hydrogen and moderately warm water, used at intervals of four hours, these injections being followed by a solution of arsenite of copper containing one milligram (one 65th grain) to the drachm, diluted with an equal quantity of hot water.

In a week the patient was able to return to his home in a distant State, the discharge from the urethra having entirely ceased, and pain and chordee having disappeared.

The author advises the same treatment for non-specific urethritis and gleet, but as hydrozone is much stronger (two times) than the peroxide, and perfectly harmless, he gives it the preference.

In vaginitis and vaginismus this treatment is of especial value. The treatment heretofore recommended by physicians, consisting of hot vaginal douches, either with or without some alkali, as sodium bicarbonate, followed by the injection of a small quantity of peroxide of hydrogen (medicinal) in warm or cold water is superseded by the single application of a hot solution of hydrozone, one part in eight; the patient should use a fountain syringe, which should be hung upon the wall about six feet from the floor; the patient sits upon a suitable vessel and introduces the rubber tip of the hose well back into the vagina, while the labia are compressed by the disengaged hand; this allows the solution to so distend the vagina as to bring it in contact with all the diseased tissue. The injection should be repeated twice in twenty-four hours.

In uterine diseases, where the solution must be brought into contact with the endometrium, the following treatment is pursued:

The patient is placed in the dorsal position, with the hips well elevated; an ordinary dilator is employed to distend the cervix, so as to admit the nozzle of the syringe and permit the free egress of the injected fluid (a suitable return flow tube can be used to better advantage; the Fritsche's douche is the best that can be used). The injection is then made, a liberal amount of the hot medicated solution being used.

There is need of caution in chronic cases, that the effervescence which attends the destruction of the unhealthy tissue does not force some of the débris into the Fallopian tubes. This is best avoided by using a large quantity of the solution, and afterwards directing the patient to assume the upright position. The pressure thus brought to bear upon the uterus will cause the complete discharge of all débris.

A preliminary vaginal douche should always be taken, using the medicated solution, as otherwise harm might ensue by the entrance into the uterus of the vaginal secretions. The author warns against the use of the vaginal douche if the cervix be patulous, as there is an almost certain danger of the infected vaginal débris being forced into the uterine cavity. To avoid this the vagina should be cleansed by the local use of the medicated solution through the speculum.

The author believes hydrozone to be the best remedy for cystitis occurring either in the male or female. The bladder should be washed out with the solution (one to eight) a small quantity being used at first in chronic cases, owing to the painful muscular contractions following the withdrawal of the solution. The amount can be gradually increased. (A double current hard rubber catheter should always be used for that purpose.) In gonorrhea, gleet and cystitis, the local treatment is oftentimes aided by the internal administration of hourly doses of calcium sulphide one-tenth of a grain.

---

### THE SURGICAL ATTRIBUTES OF THE SOUTHERN NEGRO.

Dr. Rudolph Matas, of the Charity Hospital of New Orleans, has made a very thorough statistic study of the records of that hospital as to the surgical peculiarities of the negro of Louisiana. His research, which covers a period of ten years ending 1894, has warranted the conclusion that the race of the patients plays little or no part in the prognosis of surgical operations, except in so far as it is influenced by degenerative tendencies due to the hygienic, social and moral environment. He holds the opinion that the colored man of his section is at nearly all points, whether anthropologically, physiologically and pathologically, different from his original African ancestors and from his uncivilized brothers on the west coast of Africa of the present generation. A residence of nearly three hundred years in the Southern States of North America, in contact with the white man and under the influence of civilization, has produced a marked change in the mental and physical organization of the negro. This change is evidently due to the combined influences of acclimatization and adaptation to surroundings, and especially miscegenation with the white race. The *International Journal of Surgery* weighs the argument of Dr. Matas and with him concedes that the general morbidity and mortality of the colored race was less than that of the white population in the South, during the whole period of slavery and up to emancipation; but concludes that since the colored race has been thrown upon its own resources, its morbidity and mortality have enormously increased, and are now much greater than those of the white population. While the more typical African diseases are rapidly disappearing, the general liability of the negro to the common diseases of this country is rapidly increasing, so that many immunities which he formerly enjoyed have been lost, and new predispositions to disease have been acquired. There are no conditions, continues the author, which prevail exclusively in the colored race, any more than there are diseases which prevail exclusively in the white race. The differences, pathologically speaking, that do exist between the white and colored population, lie only in their relative predispositions to some of the diseases that prevail in this country, and in their relative immunity from others. When viewed from the purely surgical operative standpoint, the white and colored races are practically alike, especially when individuls of both races, taken from the same social environment, are compared. There are no apparent differences between the races on the operating-table; the same technique applies to both equally as well, and often, especially in the matter of resistance to shock, the negro appears to better advantage than the white man. In the general and local reactions of the tissues to infection, there are some differences in the races. It is in the histogenetic tendencies of the tissues that we find the real surgical contrast between them. If we are to judge from this alone, the colored race reveals in this last particular a marked tendency to degeneration.—*Jour. Am. Med. Asso.*

### A NATIONAL HEALTH BOARD.

In the year 1891 the American Medical Association appointed a committee for the purpose of securing the passage of an act of Congress creating a National Health Board, with a Secretary of Health, the latter to be appointed by the President and to be a member of his Cabinet.

At the meeting of the association in 1896 this committee reported that it was impossible to attempt to secure the passage of an act creating a Cabinet officer, and recommended the passage of a bill creating close co-operation between the Marine Hospital Service—in itself a department of public health—and the various State and Territorial Boards of Health. The report was accepted and the recommendations adopted. Accordingly a bill was drafted by the committee, but it was impossible to get Congress, during its last session, to act upon the measure.

At the 1897 meeting of the association the committee was enlarged and made to include a representative from each State and Territory, with instructions to use every effort to get the bill before Congress.

Thus the matter stands after seven years of labor and thought. It now remains to be seen whether Congress can be prevailed upon to take up, discuss and pass upon this measure. We fear that the committee has a very difficult task set for it. Our staid and deliberate Senate is slow in making radical changes in the government, and doubtless would be especially so when the creation of a new department is involved. The formation of such a department would be a gigantic undertaking, and upon first thought our ideas as to its workings are vague and cloudy. One thing is certain, however; the introduction of the bill into Congress would bring about a free discussion in the medical and lay press. It would elicit the opinions of a large number of scientific thinkers and workers; the legal difficulties, if any existed, would be pointed out, and it would soon be ascertained whether the undertaking be feasible and necessary, or otherwise. In the institution of any department of government discussion is indispensable, and outside the American Medical Association there has been but little, as far as we can learn.

The measure is entitled "A Bill to Establish a Department of Public Health, and to Define its Duties." It is of considerable length, consisting of thirty-six sections. Omitting the details incident to the organization, management and minute duties of such a body, which are embodied in the bill, its most prominent provisions are:

*First.* That there shall be established a Department of Public Health, the duties of which shall be to collect and diffuse information upon matters affecting the public health, including statistics of sickness and mortality in the several States and Territories; the investigation by experimental and other mothods of the causes and means of prevention of disease; the collection of information with regard to the prevalence of infectious, contagious and epidemic diseases, both in this and other countries; also the causative and curative influence of climate upon the same; the publication of the information thus obtained in a weekly bulletin; the preparation of rules and regulations for securing the best sanitary condition of vessels from foreign ports, and for prevention of the introduction of infectious diseases into the United States, and their spread from one State into another, which rules, when approved by the President of the United States, shall, in so far as they are consistent with the existing laws, be adopted and enforced as quarantine regulations at the various ports of entry in the United States, and so far as applicable to interstate commerce to prevent the spread of disease from one State or Territory into another or the District of Columbia, be, and become, additional regulations thereof; the advising and informing the several departments of the government on such questions as may be submitted by them to it, or whenever, in the opinion of the Department, such advice and information may tend to the preservation and improvement of the public health.

*Second.* That the Department of Public Health shall be under the control and management of a Commissioner of Public Health; said Commissioner of Public Health shall be appointed by the President of the United States, by and with the advice and consent of the Senate.

*Third.* That the department of Public Health hereby created shall succeed to all the powers and duties now and heretofore conferred upon the Marine Hospital Service.

*Fourth.* That said department shall also, by coöperation with the proper health authorities of foreign nationalities and municipalities, endeavor to extend to the United States a reliable system of international notification of the existence and progress of such diseases as cholera, yellow fever, typhus fever, smallpox, bubonic plague, or any other dangerous or contagious disease which may, in the judgment of the department, seem advisable to consider.

*Fifth.* That the department shall, when in its judgment it may deem it necessary and proper, make such additional rules and regulations as are necessary to prevent the introduction of infectious and contagious diseases into the United States from foreign countries, or into one State or Territory, or the District of Columbia, from another State or Territory or the District of Columbia.

*Sixth.* That it shall be unlawful for any merchant ship or other vessel, from any foreign port or place, to enter any port of the United States, except in accordance with the provisions of this act.

*Seventh.* That any vessel, at any foreign port, clearing for any port or place in the United States, shall be required to obtain from the consul, vice-consul, or other consular officer of the United States at the port of departure, or from the medical officer, where such officer has been detailed by the President of the United States for that purpose, a bill of health in duplicate, in the form prescribed by the Department of Public Health.

*Eighth.* That whenever the evidence shall appear conclusive to the President of the United States that cholera, yellow fever, typhus fever, typhoid fever, smallpox, diphtheria or other plague, exists in any State or Territory, or in the District of Columbia, to such an extent that there is great danger of the spread of such disease into other States, Territories, or the District of Columbia, by means of vessels and vehicles engaged in the transportation of goods, passengers, and the United States mail, by land and water, or by persons traveling, on foot or otherwise, he is hereby authorized to call together the Commissioner of Public Health and advisory council to take such action as may be necessary to prevent the spread of such disease from one State or Territory into another, or from any State or Territory into the District of Columbia, or from the District of Columbia into any State or Territory.

*Ninth.* That all other acts or parts of acts, inconsistent herewith, or repugnant to the provisions of this act are hereby repealed.

We regret that a limited space prevents us from quoting the provisions of the bill more extensively, but trust that enough has been given to show the scope and power of such a governmental department.

There is no doubt whatever that the United States are greatly in need of an organized body, with the authority of the government behind it, to protect the sanitary, quarantine and health interests of their people; but the question is, Can the workings of such a body be adjusted so as not to conflict with an admirable Marine Hospital Service? It ought not to be impossible.

The *Medical Record* of October 30, 1897, quotes from a paper by Dr. Warren E. Anderson of Pensacola, formerly a member of the Florida Board of Health. Dr. Anderson favors the creation of a National Health Board, with essentially the same powers extended in this bill. He says "that a failure on the part of the Federal Congress to provide the citizen with adequate protection against the invasion of imported disease, and the prevention of the recurrence of such scenes as are being enacted along the lower Mississippi Valley and the Gulf coast, would be regarded as a national calamity."

The Promoters of the cause should be heard by Congress, and when once the bill is presented, it should be given the consideration that its great importance entitles it to. If destruction to life, property and business interests, such as referred to by Dr. Anderson, can be avoided by legislative enactment, and we believe it can, then it is the duty of Congress to enact the laws necessary to do it. If a National Health Board would be the means, let us have it without unnecessary delay.—*Albany Med. Annals.*

---

"It costs two hundred dollars to have a frozen toe amputated in Alaska." "Well, if you go, you would better have yours cut off before you start."—*Chicago Record.*

### THE WOODBRIDGE TREATMENT OF TYPHOID FEVER.

A correspondent asks us to publish the Woodbridge method of "aborting" typhoid fever.

The treatment is essentially one of intestinal antisepsis. Formula number one is as follows:

| | | |
|---|---|---|
| ℞ | Resini podophylli | gr. $\frac{1}{960}$ |
| | Hydrargyri chloridi mitis | gr. $\frac{1}{16}$ |
| | Guaiacol carbonatis | gr. $\frac{1}{16}$ |
| | Menthol | gr. $\frac{1}{16}$ |
| | Eucalyptol | q. s. |
| | Misce et fiat triturate No. i. | |

Sig.: One every fifteen minutes during the wakeful period of the first forty-eight hours, or until eighty or one hundred have been taken.

Large potations of sterilized carbonated water are recommended.

No. 2. At the end of twenty-four hours begin with:

| | | |
|---|---|---|
| ℞ | Resini podophylli | gr. $\frac{1}{960}$ |
| | Hydrargyri chloridi mitis | gr. $\frac{1}{16}$ |
| | Guaiacol carbonatis | gr. $\frac{1}{4}$ |
| | Menthol | gr. $\frac{1}{16}$ |
| | Thymol | gr. $\frac{1}{16}$ |
| | Eucalyptol | q. s. |
| | Misce et fiat tablet No. i. | |

Sig.: Give one every fifteen minutes with No. 1 until five or six free evacuations of the bowels have been procured, during this and the following day.

About the third or fourth day Dr. Woodbridge gives:

| | | |
|---|---|---|
| ℞ | Thymol | gr. i. |
| | Guaiacol carb | gr. iii. |
| | Menthol | gr. ss. |
| | Eucalyptol | m. v. |
| | Misce et fiat capsule No. i. | |

Sig.: Give one every three hours until temperature has been normal for at least three days.

For children the same medicinal treatment is instituted in smaller doses.

---

ABSORBENT cotton does not make good padding for splints. Ordinary cotton-batting is far more elastic and will not gradually pack together by absorbing moisture from the skin.—*Ex.*

### COMPENSATION OF MEDICAL WITNESSES IN COURT.

In the supreme court of Illinois a decision was handed down which is of great interest to physicians and others who may be called upon to give expert testimony in court. It is held by many professional men that they cannot be compelled to give a professional opinion without a reasonable compensation, and in support of this they quote that portion of the constitution which declares that no person shall be deprived of property without due process of law. The case in which the decision was handed down is an appeal of Dr. J. Norman Dixon from a judgment rendered against him by Judge Creighton, of the Sangamon circuit court, some time ago, in which it was agreed a test should be made. Dr. Dixon was called to give expert testimony in a damage suit for personal injury against the city of Springfield. He refused to testify unless he was paid a professional fee of $10. Judge Creighton fined him $25 for contempt of court, and the case was appealed to the appellate court, where Judge Creighton's judgment was affirmed. The case then went to the supreme court, where the judgment was again affirmed. The briefs in the case were prepared by State's Attorney James M. Graham, and the ground taken was that professional knowledge of the kind required in expert testimony is not properly within the meaning of the constitutional provision, as it cannot be given away nor alienated from the person possessing it, and that in the exercise of the right of the court to summon witnesses and compel them to testify no distinction can be made between kinds of knowledge without endangering the ends of justice. This view of the case was upheld by the appellate court, and now the supreme court affirms it. Judge Creighton's decision attracted general attention when it was announced and called forth the strongest condemnation from members of the medical profession. However, he is now upheld by the highest judicial tribunal in the State.—*Jour. Am. Med. Asso.*

---

### THE WIDAL TEST FOR TYPHOID FEVER.

Dr. Gilman Thompson has collected data from five New York hospitals relative to the Widal serum test for typhoid fever. In the *Medical News,* October 30th, he publishes the following conclusions:

1. While the test is positive in the large majority of cases of enteric fever, and negative in the greater number of other diseases, there is a margin of error of eleven to twelve per cent. on each side of what might be called the normal line, between cases in which the test fails where it ought to succeed and succeeds where it ought to fail, in order to make it of real clinical value.

2. This total of twenty-three per cent. of possible error includes, unfortunately, just those cases in which there is the greater doubt upon the purely clinical side.

3. As a genuine diagnostic aid the test has about the value of the Diazo reaction in typhoid urine, or the study of leucocytosis in pneumonia; that is, it is confirmatory in connection with appropriate symptoms, but misleading if positive reliance be placed upon it.

4. The fact that an expert bacteriologist is required to make the test is offset by the ease of transportation of specimens of dried blood which long retain the power of reaction, and it is greatly to be hoped that a further possible improvement in technique may place this most ingenious test upon a firmer practical basis than can at present be claimed for it.

---

### TREATMENT OF ACUTE TONSILLITIS.

The following plan of procedure is recomended by Grasset in the treatment of acute tonsillitis:

Milk is given every two hours, day and night.

Every two hours a teaspoonful of the following mixture is to be administered:

| | | |
|---|---|---|
| ℞ | Potassium chlorate | 16 grains. |
| | Water | 1 fluid ounce. |

During the day the throat should be gargled frequently with a hot infusion, or with the following combination:

| | | |
|---|---|---|
| ℞ | Sodium naphtholate | 5 grains. |
| | Cocain hydrochlorate | 1½ grains. |
| | Syrup | 1 fluid ounce. |

The throat should be painted three or four times during the day with

| | | |
|---|---|---|
| ℞ | Glycerin | 4½ grains. |
| | Borax | 1 dram. |

If the local condition should become alarming silver nitrate, 1–10, should be applied topically, and the throat sprayed with carbolized water or a solution of phenosalyl, 1–100. If the pain is severe, especially on deglutition, the throat should be painted three or four times a day with

| | | |
|---|---|---|
| ℞ | Cocain hydrochlorate | 8 grains. |
| | Glycerin | 1 fluid ounce. |

The treatment may be begun with ipecac if this drug has not already been given. The patient should be isolated and separated, especially from children and those susceptible to tonsillitis.

The latter may receive by spray morning and night, two fluidrams of the following solution:

| | | |
|---|---|---|
| ℞ | Cocain hydrochlorate, Carbolic acid, each | 7½ grains. |
| | Glycerin | 6 fluidrams. |
| | Water, sufficient to make | 1 pint. |

Or the throat may be painted daily with

| | | |
|---|---|---|
| ℞ | Tincture of iodin, Glycerin | equal parts. |

By the mouth sulphur and arsenic are alternated from month to month. For twenty days a half-glass of some sulphurous water is taken each morning on an empty stomach. This is also used as a gargle. The patient then rests for ten days. At the expiration of this time he takes twice daily, for the next twenty days, tablespoonful of

| | | |
|---|---|---|
| ℞ | Sodium arsenate | 1½ grains. |
| | Distilled water | 10 fluid ounces. |

Then again the patient rests for ten days, and the alternation is thus kept up. Tobacco and alcohol are to be abstained from. Chilling, and association with cases of tonsillitis are to be avoided. If possibie, a visit should be made annually to some sulphur resort. In case of frequent recurrence of attacks, recourse should be had to the galvano-cautery, or amygdalotomy, according to the size of the tonsils.—*Medical News.*

### FOR ACUTE ALCOHOLISM.

The following combinations are recommended:

℞ Spt. ammon. aromat.......................... ʒ ii.

Tinct. camphoræ.......................... ʒiss.

Tinct. hyoscyami.......................... ʒiiss.

Spts. lavandulæ co.......................... q. s. ad ℥ ii.

M. Sig. One teaspoonful every hour.

When acute symptoms have been relieved the following may be substitued:

℞ Pulv. capsici.......................... gr. xxiv.
Quininæ sulph.......................... gr. xxxvi.
M. Ft. cap. No. xii. Sig. One capsule before each meal and continue for several days.

Should insomnia be an element, administer the following:

℞ Sodii bromidi.......................... ℥ ss.
Chloral. hydrst.......................... ʒ iiss.
Syr. aurantii cort.......................... ℥ ss.
Aquæ.......................... ad. ℥ iv.
M. Sig. A teaspoonful at bedtime and repeated during the night if necessary.

—*N. Y. Med. News.*

---

### PLEURISY WITH EFFUSION.

Among measures to promote absorption the best, in my own experience, is the following combination:

℞ Potass. iodidi.......................... ʒ i.
Syr. feri iodidi.......................... ʒ ii.
Syr. sarsap. co.......................... ℥ i.
Essence pepsin, q. s. ad.......................... ℥ ij.
M. Sig. Teaspoonful every four hours, diluted. The dose to be doubled at the end of four days if well borne by the stomach.

I have employed this formula in more than fifty cases with very good results.—*Dr. J. M. Anders, Practice of Medicine.*

---

### FOR ALCOHOLIC MANIA.

℞ Sodii bromid.......................... ℥ j.

Tr. capsici.......................... ʒ j.

Tr. digitalis.......................... ʒ ss.

Elix. simplicis.......................... q. s. ad ℥ ij.

M. Sig. ʒ j every two or three hours in water.

—*J. M. Anders, M.D.*

### THE ADMINISTRATION OF COD-LIVER OIL.

Bricemoret (cited in the *Journal des practiciens* for October 23d) recommends the following formula:

| | | |
|---|---|---|
| ℞ | Cod-liver oil | 15 ounces. |
| | Syrup of Tolu | 7½ ounces. |
| | Tincture of Tolu | 12 drops. |
| | Essence of cloves | 2 drops. |

M. S.: A tablespoonful two or three times a day, the bottle being well shaken before the dose is poured out.

—*N. Y. Med. Jour.*

---

"IF I were ill most assuredly I would not seek the assistance of a chemist, or of a physiologist, and medicine is not to be learned from the books of Claude Bernard or of Pasteur. Clinical instruction is necessary, such as long observation of patients alone can furnish. Prophylaxis, diagnosis, prognosis, therapeutics are not to be learned in scientific books. Something else is necessary—observation, long, patient observation, the old Hippocratic observation, without which there can be no good physician. Young students must be guided in the examination of patients by experienced practitioners, and no one, I presume, would be guilty of the folly of proposing to replace the clinical ward by the laboratory."—*Richet.*

---

ONE of the dangers in amputation of the breast is pneumonia due to exposure at the time of operation. The natural protection by the mammary gland and adipose tissue is taken away, and a moist evaporating surface very close to the lung invites subsequent congestion. Much danger may be avoided by covering all parts of the wound not at the time the actual seat of operation, with hot, wet towels; and frequently changing them as they become cool. Hot water may also be occasionally poured over the protecting towels.—*International Jour. of Surg.*

---

"MISTER," said the small boy to the chemist, "give me another bottle o' them pills you sold father day before yesterday."

"Are they doing him good?" asked the chemist, looking pleased

"I d'no whether they're doin' father any good or not, but they're doin' me good. They just fit my new air-gun."—*Ex.*

